# A Neuropsychological Approach to Auditory Verbal Hallucinations and Thought Insertion - Grounded in Normal Voice Perception

**DOI:** 10.1007/s13164-015-0270-3

**Published:** 2015-06-04

**Authors:** Johanna C. Badcock

**Affiliations:** Centre for Clinical Research in Neuropsychiatry, School of Psychiatry and Clinical Neurosciences, University of Western Australia, Crawley, 6009 Western Australia

## Abstract

A neuropsychological perspective on auditory verbal hallucinations (AVH) links key phenomenological features of the experience, such as voice location and identity, to functionally separable pathways in normal human audition. Although this auditory processing stream (APS) framework has proven valuable for integrating research on phenomenology with cognitive and neural accounts of hallucinatory experiences, it has not yet been applied to other symptoms presumed to be closely related to AVH – such as thought insertion (TI). In this paper, I propose that an APS framework offers a useful way of thinking about the experience of TI as well as AVH, providing a common conceptual framework for both. I argue that previous self-monitoring theories struggle to account for both the differences and similarities in the characteristic features of AVH and TI, which can be readily accommodated within an APS framework. Furthermore, the APS framework can be integrated with predictive processing accounts of psychotic symptoms; makes predictions about potential sites of prediction error signals; and may offer a template for understanding a range of other symptoms beyond AVH and TI.

**Electronic supplementary material:**

The online version of this article (doi:10.1007/s13164-015-0270-3) contains supplementary material, which is available to authorized users.

## Introduction

Auditory verbal hallucinations (AVH) and the experience of thought insertion (TI) are commonly discussed together as characteristic or ‘first-rank’ symptoms (FRS) of schizophrenia (Schneider 1959; see Table 1). However, it is increasingly clear that these symptoms also occur in a range of other disorders and in the general population (Beavan et al. 2011; Nordgaard et al. 2008; Nuevo et al. 2013; Rössler et al. 2007). Such findings have contributed to a renewal of interest in dimensional ways of thinking about psychopathology (Insel et al. 2010), which start with the assumption that symptoms – such as AVH and TI – arise from disturbances in basic psychological and physiological mechanisms underpinning human behaviour (Badcock et al. 2014; Ford et al. 2014). It has previously been proposed that some of the characteristic features of AVH (such as the identity and location of hallucinated *voices*^1^) can be grounded in the mechanisms of human voice perception; specifically, the abnormal activation of parallel auditory pathways underlying the identification (‘what’) and localization (where) of sounds (Badcock 2010). The evidence regarding the differential properties of separable auditory processing streams has provided a valuable ‘framework’, i.e., a broad approach, for thinking about AVH (Wilkinson 2014). However, it has not previously been extended to other closely related symptoms, such as TI - yet inserted thoughts are traditionally viewed as being phenomenologically and causally related to AVH (Humpston and Broome 2015; Nelson et al. 2014). In this paper, I aim to show that an auditory processing streams (APS) framework may help us to understand the experience of TI as well as AVH, providing a common conceptual framework for both. Specifically, I suggest that a neuropsychological approach to psychotic symptoms, based on parallel auditory pathways, can help us to understand both the similarities and differences in the characteristic features of TI and AVH. I also suggest the APS framework can be united with predictive coding accounts of hallucinations and delusions (Adams et al. 2013; Fletcher and Frith 2009), providing the basis for a more integrated model of these symptoms.

**Table 1:** Definitions and examples of thought insertion and auditory verbal hallucinations

Definition	Example questions	Example descriptions (self-reports)
Thought insertion The subject experiences thoughts which are not his own intruding into his mind. The symptom is not that he has been caused to have unusual thoughts, but that the thoughts themselves are not his. [Wing et al., 1983].	Do there ever seem to be thoughts in your mind which are not your own; which seem to come from elsewhere? [DIP 20.49; SCAN 18.006] Do you ever feel as if the thoughts in your head are not your own? [CAPE #26].	“Often, in a quiet place, and all the time at night when I am alone, I experience thoughts that do not “feel” like my own. It’s like they come out of a part of my brain that is not the part that controls my “normal” thoughts and into my awareness from there. It is hard to describe.” [‘alienonite’ on Crazyboards website*]
Auditory hallucinations A sensory experience which occurs in the absence of corresponding external stimulation of the relevant sensory organ; has a sufficient sense of reality to resemble a veridical perception, over which the subject does not feel s/he has direct voluntary control and which occurs in the awake state. [David, 2004]	Have you ever heard noises or voices when there is nobody about and no ordinary explanation seems possible? [SCAN 17.001) Do you ever hear voices talking to each other rather than to you? [DIP 20.48; SCAN 17.009] Do you ever hear voices talking to each other when you are alone? [CAPE #34]	“You have to kill yourself” [‘Anna Peter’ on intervoiceonline*] “Isn’t he stupid, he never gets anything right”

*DIP* diagnostic interview for psychosis, *SCAN* Schedules for clinical assessment in neuropsychiatry, *CAPE* community assessment of psychic experience. *CIDI* composite international diagnostic interview *http://www.crazyboards.org/forums/index.php/topic/37139-thought-insertion/?hl=alienonite#entry401745 * *http://www.intervoiceonline.org/about-voices

To illustrate these ideas I begin with a brief description of the similarities and differences in phenomenology and epidemiology of AVH and TI and critique how alternative explanations based on Forward Models fare in accounting for these characteristics. I then introduce the APS framework, explore the characteristic features of AVH and TI in terms of the functional division of auditory processing streams, and conclude by outlining the implications and challenges for future research.

### Similarities and Differences in the Phenomenology of AVH and TI

At the individual level of analysis, both AVH and TI are experienced as intrusions that interrupt ongoing thoughts and events. People with these symptoms describe a sense of passivity about their experiences - “they come unasked” (Jaspers 1963, p. 123), along with a diminished sense of control that helps distinguish these experiences from ordinary verbal thought (Hoffman et al. 2008; see Table 1). Furthermore, in the majority of cases both AVH and TI are experienced as intrusions generated by an external identity; that is, they are perceived as a *voice* or a thought which is not regarded as one’s own, but seems to belong to someone else (though “own voice” hallucinations also occur; e.g., Corstens and Longden 2013). Despite these phenomenological similarities AVH and TI also differ in important ways. Most notably, AVH are typically described as having a range of auditory sensory qualities (e.g., voices varying in loudness, tone and clarity; McCarthy-Jones et al. 2014) which inserted thoughts usually lack. However, when examining first-person reports it is also clear that at least some hallucinated *voices* are described as “soundless” or “inaudible”, while some inserted thoughts are cast as internal *voices* (Woods et al. 2014). Therefore, the supposed distinction in auditory-sensory quality between AVH and TI gives way to a degree of overlap in audibility (Humpston and Broome 2015). A further difference arises in terms of the location and manner in which these symptoms are experienced. In particular, people with TI believe that “somebody is *putting* thoughts *into my head*” (emphasis added; Wilkinson 2014) – that is, the experience is located internally or at least arriving in personal space. In contrast, it has long been recognized that people with AVH report hearing voices both inside and outside the head. Indeed, a recent phenomenological survey of 199 psychiatric patients showed that the location of AVH was equally likely to be reported in internal or external space, or both, during the last episode (McCarthy-Jones et al. 2014). It is clear, therefore, that a common neuropsychological framework which aims to account for both AVH and TI must be able to accommodate both the overlap and the differences between these experiences.

### Similarities and Differences in the Epidemiology of AVH and TI in Psychosis

At the population level, epidemiological data reveal both differences (in prevalence) and similarities (or co-occurrence) in AVH and TI that also points to the presence of some separate, and some shared functional mechanisms. For example, in a now classic study in the field, Sartorius and colleagues found that approximately 70 % of patients with schizophrenia reported AVH on the Present State Examination, whilst only 25–42 % experienced TI, with variation depending on the country of origin (Sartorius et al. 1986; see also Shinn et al. 2012). More recent data drawn from the Survey of High Impact Psychosis, 2010 (SHIP; Morgan, personal communication; see Table 2), which is based on a representative sample of patients in public treatment services in Australia (Morgan et al. 2012, 2014), shows a similar discrepancy in prevalence between TI and AVH, regardless of the specific diagnostic category assessed. Confidence in these differences relies on the psychometric properties of the assessments used, the interviewers’ skills to elicit and judge symptoms, and the ability of individual patients to talk about their experiences. With these limitations in mind, these data seem to indicate that hallucinated *voices* are much more common than inserted thoughts, which suggests that their underlying mechanisms differ.

**Table 2:** Prevalence of thought insertion and auditory verbal hallucinations in psychosis in the Survey of High Impact Psychosis

	Schizophrenia *N* = 857	Schizoaffective disorder *N* = 293	Bipolar disorder with psychosis *N* = 319	Depressive psychosis *N* = 81	Delusional & other non-organic psychoses *N* = 92	*Other psychosis *N* = 183	Total *N* = 1825
Symptoms^a^	N (%)	N (%)	N (%)	N (%)	N (%)	N (%)	N (%)
Thought insertion (Present state)	128 (14.9)	58 (19.8)	16 (5.0)	12 (14.8)	7 (7.6)	6 (3.3)	227 (12.4)
Thought insertion (Lifetime)	279 (32.6)	119 (40.6)	65 (20.4)	21 (25.9)	14 (15.2)	23 (12.6)	521 (28.5)
Auditory verbal hallucinations (Present state)	334 (39.0)	97 (33.1)	33 (10.3)	16 (19.8)	17 (18.5)	15 (8.2)	512 (28.1)
Auditory verbal hallucinations (Lifetime)	640 (74.7)	208 (71.0)	128 (40.1)	38 (46.9)	44 (47.8)	49 (26.8)	1107 (60.7)

^a^ Symptoms assessed with the Diagnostic Interview for Psychosis: thought insertion (DIP item 20.49); auditory verbal hallucinations (DIP items 20.46, 20.47, 20.48). *Other psychosis: Screen-positive for psychosis incl. severe depression but did not meet full criteria for ICD-10 psychosis. Data provided with permission from Prof. Morgan (derived from Morgan et al. 2012)

Conversely, AVH and TI often co-occur, which tends to imply that their underlying mechanisms overlap. Although, factor analytic methods have often been employed to examine how symptoms aggregate, the best level of analysis to capture symptom-mechanism links is still unknown. Thus, AVH and TI are often considered together, in a ‘positive symptoms’ factor (e.g., Ventura et al. 2010), but sometimes considered apart (i.e., in separate factors; e.g., Heering et al. 2013), consistent with shared or distinct functional processes, respectively. Capturing this complexity, Peralta and Cuesta proposed a hierarchical structure for psychotic symptoms. At the first level of their analysis they identified 13 inter-correlated primary factors. However, a further factor analysis of these first-order factors yielded five uncorrelated “higher-order” factors, which accounted for most of the symptom covariance (63.5 %) (Peralta et al. 2013). Importantly, in this hierarchical model, TI retained higher loading as a first-order factor whilst AVH formed a higher-order factor, which suggests the experience of hearing *voices* indexes a more general set of features (and corresponding set of mechanisms) whilst TI is more specific. Of course, symptom co-occurrence can sometimes be determined from phenomenological or epidemiological survey data as well – though is often lost through symptom pooling. For example, Nayani and David (1996) observed that 39 % of patients with AVH also reported TI, whilst more recent data from the SHIP (Morgan et al. 2012) shows that the risk of TI is increased over 6-fold among those with AVH, relative to those without (present state OR = 6.65, 95 % CI: 4.94 – 8.97; lifetime OR = 3.17, 95 % CI: 2.50 – 4.01) (see supplementary Table 1). Overall, the challenge that emerges from this data is whether a common conceptual framework can explain why the experience of hearing *voices* is more common than, yet increases the risk for, TI in psychosis.

### Similarities and Differences in the Epidemiology of AVH and TI in the General Population

The literature discussed so far relates to the expression of AVH and TI after illness onset. However, recent studies suggest that the dynamic co-occurrence of hallucinations and delusions in the general population is particularly important in the transition to psychosis (Nuevo et al. 2013; Smeets et al. 2013). The experience of AVH in non-clinical samples is now well-recognized and intensively studied. For example, population based studies have shown that hallucinated *voices* are relatively common during childhood and the early teenage years, reported in 21–23 % of individuals. The majority of these symptoms appear to be transitory, with prevalence in older adolescents falling to around 7 % (Preti et al. 2014), and may simply reflect normal fluctuations in cognitive development (Badcock and Hugdahl 2014; Kelleher et al. 2012). However, for some, hallucinations persist and carry an increased risk of transitioning to a range of psychopathologies (Fusar-Poli et al. 2013). In comparison, TI-like experiences in non-clinical groups have been given much less attention. Nonetheless, studies using self-report measures suggest that TI also occurs in the general community including, but not exclusive to, young adults at ultra-high risk for psychosis (Fonseca-Pedrero et al. 2012; Mossaheb et al. 2012; Wiles et al. 2006). For example, Mossaheb and colleagues found that approximately 43 % of ultra-high risk subjects reported the presence of TI on the Community Assessment of Psychic Experiences questionnaire (see Table 1), compared to around 37 % of those assessed as not at risk (Mossaheb, personal communication), whilst Rössler and colleagues reported the four-week prevalence rate of TI (defined as “having thoughts that are not your own”) in 20–21 year olds from the general community was 22.6 %, compared to 3.2 % for auditory hallucinations (Rössler et al. 2007). These data appear to indicate some important age-related differences in the experience of AVH and TI in the general community, which merit further attention in longitudinal studies. Of course, another possibility is that positive endorsement of TI or AVH in these studies reflects a qualitatively different kind of experience than in psychosis, though this concern is partly mitigated by cross-validation of self-report tools with interview-based measures (e.g., Konings et al. 2006; Mossaheb et al. 2012). On the other hand, the evidence seems to show that healthy individuals with hallucinations are at significantly greater risk of delusions than those without, with TI much more likely to co-occur with AVH than other types of delusion, (i.e., this specific combination of symptoms is unlikely to be simply a random event). Indeed, Smeets and colleagues proposed that this particular confluence of symptoms could represent a critical phase in the earliest stages of transition to psychosis (Smeets et al. 2013). If correct, this implies that the dynamic co-occurrence of AVH and TI is not simply a general marker of illness severity, but a specific index of the likelihood of illness progression. In turn, this would also suggest that impairments in the underlying neuropsychological mechanisms common to AVH and TI play a key role in the transition to psychosis.

To summarize, a direct comparison of hallucinated *voices* and inserted thoughts reminds us that they share some characteristic features (e.g., a sense of diminished control and intrusion of external identity), but also differ in others (e.g., in perceived location). The evidence also suggests that hallucinated *voices* are more common than, though strongly associated with, inserted thoughts, and together they may play an important role in the development of psychosis. Overall, these differences and similarities suggest there may be partial, but not complete, overlap in the underlying mechanisms of AVH and TI – which I turn to next.

## The Forward Model Framework

The most influential neurocognitive account of AVH and TI suggests these symptoms result from a dysfunctional self-monitoring mechanism. Ordinarily, it is assumed, the process of self-monitoring allows us to distinguish between self-generated and externally-generated stimuli. At the neural level, self-monitoring has been linked to a ‘forward model’ which allows us to compare the predicted and observed outcomes of a movement. If these match, then we can assume the movement was self-generated. According to this account, first rank symptoms of schizophrenia occur when this comparator mechanism is dysfunctional, leading self-generated stimuli to be experienced as arising from an external cause (Feinberg 1978; Frith 1992). In the case of AVH and TI, what has been proposed is that both of these symptoms could be explained in terms of a failure to properly monitor the movement entailed in the production of inner speech, which is then misattributed to an external source (Jones and Fernyhough 2007). However, this explanation has been subject to a number of criticisms (see e.g., Waters et al. 2006; Wilkinson 2014). These problems are best illustrated here by thinking about the similarities and differences in these symptoms described above. For example, if aberrant monitoring of one’s own inner speech accounts for the misattribution to an external identity in AVH and TI then why do many people report experiencing the *voice* or thoughts of someone else, i.e., another specific person (e.g., the thoughts of a man called Pete, or the voice of a past abuser). How does this ‘transformation’ occur? Furthermore, if misattribution results from an abnormality in self-monitoring then what additional mechanism accounts for the sense of diminished control common to AVH and TI? On the other hand, if we treat both AVH and TI as the result of a single mechanism involving misattributed inner speech then how do we explain their differences in perceived location and prevalence, for example? In sum, self-monitoring theory falls short as an adequate account of the characteristic features of AVH and TI.

Since its initial description, the comparator model has been elaborated and refined, with attention now focused on the computations involved in predicting *all* (rather than just self-produced) stimuli, within a “predictive processing framework” (PPF; Clark 2013; Frith 2012).^2^ In the PPF, a prediction is a prior expectation (of the state of the world) based on stored knowledge, which is used to construct the incoming sensory signal ‘from the top down’, whilst sensory feedback provides the basis on which the prior should be updated. Within these more integrated forward models the emphasis is on precision and minimization of prediction error signals – “the deviation between the state one expects to experience and what is actually experienced” (Griffiths et al. 2014, p. 439) - which have long been considered critical for normal cognition. This interest is now being matched by growing speculation that abnormal prediction coding is implicated in the positive symptoms of psychosis (Adams et al. 2013; Fletcher and Frith 2009; Griffiths et al. 2014). Wilkinson (2014), for example, has argued that a PPF can account for auditory hallucinations based on external stimuli (e.g., hypervigilance hallucinations, Dodgson and Gordon 2009), as well as internal stimuli (such as inner speech). For the latter, Wilkinson suggests that the distinct auditory phenomenology of AVH arises from imprecise predictions (in speech processing areas) about the acoustic consequences of inner speech, which results in a misperception that someone else is speaking. Similarly, if hallucinations are considered to arise from a reduction in the precision of priors (which are based on stored knowledge) then a PPF can also be united with the idea that AVH involve a failure to suppress memories that are irrelevant to ongoing events (Badcock and Hugdahl 2012; Badcock et al. 2005). In general, however, the current line of thinking is that AVH (and therefore potentially TI as well) may arise from an imbalance between perceptual expectations (i.e., heightened predictions) and actual sensory input, related to deficiencies in processing prediction error (Friston 2005; Nazimek et al. 2012). Moreover, abnormal prediction error signaling is likely to contribute to deficiencies in associative learning (e.g., learning to distinguish between predictive and non-predictive/irrelevant cues) and the formation of maladaptive beliefs in individuals with these symptoms, consistent with previous evidence (Corlett and Fletcher 2015; Morris et al. 2013). Nonetheless, a number of challenges remain with predictive coding accounts of psychotic symptoms in general, and of AVH and TI in particular. For example, there needs to be some account of why specific symptoms differ from one person to another (Frith 2012) or, indeed, why TI and AVH differ at all. If (some) hallucinated *voices* and all inserted thoughts arise from imprecise predictions of inner speech then how do we account for their differences in auditory phenomenology (the gradient in auditory sensory qualities and distinction in perceived location), and why are TI rarer? Others have argued that a “mere failure of prediction is not sufficient to generate a delusion” (p. 53, Frith 2012) - a belief that an unusual sensory experience is real (see Coltheart et al. 2011; Griffiths et al. 2014). If prediction errors signal the intrusion of perceptual anomalies or the onset of intrusive thoughts (i.e., the content of AVH and TI) then a second mechanism is required to explain why the experience is believed to be real – a point which I will return to later. Alternatively, it’s possible to think of prediction error signals being implemented at successively higher levels of the cortical hierarchy (i.e., a single core abnormality; Fletcher and Frith 2009), but the level or site in the cortical hierarchy that is relevant to specific symptoms (such as AVH and TI) remains underspecified. I suggest this limitation might be (partly) addressed by embedding predictive coding mechanisms within an APS framework.

## An Auditory Processing Stream Framework

If AVH and TI have their origins in normal cognitive and neural mechanisms, then our explanations of how they arise need to be grounded in the best available models of human perception and cognition. AVH, for example, are usually conceived as the abnormal perception of voices; consequently current models of voice perception provide a useful overarching framework for understanding both the intra-(cognitive and neural) and inter-(social) individual mechanisms driving the onset and diversity of hallucinated *voices* in psychotic and non-psychotic groups (Badcock 2010; Badcock et al. 2014). Accordingly, the perception of voice has been shown to entail more than just speech/language; it involves a wealth of socially important information about the identity, affect and location of a speaker (Belin et al. 2011). Emerging evidence indicates that these different types of input are processed in voice-sensitive regions of auditory cortex – the “temporal voice areas” - and thereafter in parallel, and hierarchically organized, ventral (anterior superior temporal to inferior frontal) and dorsal (posterior superior temporal, inferior parietal, and superior frontal) processing streams. These pathways subserve the two main functions of hearing, identification (sound-to-meaning mapping) and localization (sound-to-motor mapping) and represent two separate sources of forward prediction (Alho et al. 2014; Belin et al. 2011; Hickok 2012; Latinus and Belin 2011; Leavitt et al. 2011; Rauschecker 2011). Typically, the input in these pathways is seamlessly integrated into memory, as a coherent perceptual whole; however, their segregated nature means that each kind of vocal information (words, identity, location, etc.) can be disturbed somewhat independently of the others. Importantly, empirical evidence suggests that the phenomenological diversity of AVH (e.g., identity of *voices* and their localization in space) can be understood (at least in part) in terms of the specific pattern of dysfunction, at different hierarchical levels, within and between these auditory processing streams (Badcock 2010; Badcock and Chhabra 2013; Chen et al. 2013; Looijestijn et al. 2013). Accordingly, the similarities and differences in AVH and TI noted above, could be conceived as emerging as a result of partial, but not complete, overlap of dysfunction in these auditory processing streams – as shown in simplified form in Table 3. The following sections will elaborate on this idea.

**Table 3:** A multi-dimensional approach to AVH and TI based on auditory perception

Spatial Location (Dorsal)	Identity (Ventral)	
	Self	Other
Internal location	Perception of voice	Thought insertion AVH (inside the head)
External location	Gedankenlautwerden	AVH (outside the head)
	‘Own thought AVH’	
	Thought broadcast	

### Abnormal Activation of Auditory Processing Streams: Intrusions and Diminished Control

The fundamental premise of the APS framework is that dysfunctional activation of auditory processing streams co-opts the same neural resources used to process “real” external stimuli, which *intrudes* into ongoing mental events and becomes confused with reality. This general formulation can readily account for the shared sense of passivity in AVH and TI (see section 1.1). It can also account for experimental evidence of an increase in intrusive cognitions in people with AVH and TI, both in the presence (Brebion et al. 2010; Brebion et al. 2012; see also Marzillier and Steel 2007) and the absence of a specific task (Lobban et al. 2002; Morrison and Baker 2000). For example, both healthy individuals prone to hearing *voices* and patients with ‘thought interference’ (e.g., thought insertion) have higher scores on self-report measures of intrusive thoughts in daily life (e.g., There are thoughts that keep jumping into my head) compared to controls (Linney and Peters 2007; Smailes et al. 2015; Varese et al. 2010; Vellante et al. 2012).

Although intrusions are a central feature of AVH and TI, the underlying causes of abnormal activation in auditory pathways are still a matter of debate. Some have proposed, for example, that AVH begins with aberrant cortical activity in the medial temporal lobe and then propagates to temporal and parietal auditory streams, making the experience sensory (Jardri et al. 2011). Alternatively, AVH and TI seem to involve a tendency to mentally *wander away* (e.g., from the task at hand) and *wander towards* something else (e.g., a personal concern) which may point to a disturbance in the brain’s default mode network (DMN; Whitfield-Gabrieli and Ford 2012) – a major neural correlate of stimulus independent thought (Christoff 2012; Mason et al. 2007). Consistent with this proposal, hyper-activation of associative sensory (e.g., auditory) cortex has been shown to occur when the DMN is *disengaged* and correlates with the severity of hallucinations (Jardri et al. 2013). Empirical studies of TI and DMN are still missing; however, an intriguing possibility is that the similar tendency to intrusive cognitions reflects spontaneous engagement (intrusive thoughts/mind wandering) and withdrawal (AVH/TI) from DMN activity, respectively (Gerrans 2013).

Dysfunctional activation of auditory pathways can also be considered from a PPF perspective. Horga and colleagues, for example, examined sensory prediction errors in schizophrenia patients with daily AVH (Horga et al. 2014). They manipulated participants’ expectation of hearing speech by varying the probability of speech stimuli in a speech decision-making task. Functional imaging showed that patients activated a voice-sensitive region in auditory cortex when hearing *voices,* which was associated with deficient prediction error signals in the same region. In addition, this prediction error deficit was strongly associated with increased cortical activity during silence. The authors therefore concluded that deficient predictive coding could account for hyperactivity in auditory cortex that leads to AVH (Horga et al. 2014). Could deficient predictive coding also apply to TI? The results of Horga’s study showed that the magnitude of the predictive coding deficit was associated with the severity of AVH, but not other psychotic symptoms. So it’s possible that the PPF can account for hallucinations but not inserted thoughts - though this runs counter to the proposal that aberrant predictive coding underlies *all* positive psychotic symptoms (Fletcher and Frith 2009). Alternatively, given the specific response properties of voice-sensitive cortex, predictive coding errors in this particular region may result in the auditory-sensory quality of ‘hearing’ a *voice,* which usually differs (i.e., is typically absent) in TI (see Section 1.1). This proposal could be tested by employing the same decision-making task to determine if deficient predictive coding in this region is associated with TI cast as “*voices*” but not with AVH described as “soundless”.

It is important to remember that intrusive cognitions are usually met by a response in the central executive network in an attempt to reinstate control and coordinate thoughts and actions (Niendam et al. 2012). Both the tendency to intrusions and diminished sense of control shared by people with AVH and TI (see Table 1) suggests a common impairment in the ability to control and regulate mental representations - be they individual features of auditory stimuli (words, voice, identity etc.) or entire episodes in memory. Consistent with this proposal, there is mounting evidence that the presence and persistence of AVH, in psychotic and non-psychotic *voice* hearers, is associated with deficits in cognitive inhibition (reviewed in Badcock and Hugdahl 2014; El Haj et al. 2015). For example, on repeated runs of a continuous recognition task (all composed of the same set of pictures) schizophrenia patients with current AVH produce significantly more incorrect responses to distractors (false alarms) seen on previous runs than non-hallucinating patients, indicating an inability to suppress recently activated but currently irrelevant memories (Badcock et al. 2005). Correct performance on this task requires the ability to judge whether a currently active representation (a memory/thought) pertains to present reality, or not, and involves the rapid activation of posterior orbitofrontal cortex (OFC), leading Schnider and colleagues to refer to this mechanism as ‘orbitofrontal reality filtering’ (Liverani et al. 2015; Schnider 2013).^3^ Conversely, impairment of this mechanism allows irrelevant representations (memories, thoughts) to intrude into current events and be treated (believed) as real^4^ (Badcock et al. 2005; Waters et al. 2003) and could therefore provide a common functional component of TI as well as AVH (Vosgerau and Voss 2014). This prediction could easily be tested by examining the performance of patients and healthy individuals with inserted thoughts on various measures of executive/inhibitory control. However, it is important to note that an impairment in this capacity alone is not sufficient to generate hallucinations or inserted thoughts. Crucially, patients with lesions to the OFC or with intrusive, obsessional thoughts, exhibit significantly impaired performance on measures of inhibitory control, in the absence of either AVH or TI (Badcock et al. 2007; Schnider and Ptak 1999). This adds further weight to the view that the phenomenological complexity of psychotic symptoms cannot be explained on the basis of a single mechanism alone (Ford et al. 2014; Vosgerau and Voss 2014).

In sum, the similarities in AVH and TI related to intrusion and control can be easily understood within an APS framework, which unites a range of behavioural and neural evidence on these symptoms. Furthermore, predictive coding accounts can be embedded into this framework leading to testable predictions about the role of basic voice processing mechanisms in the auditory-sensory quality of AVH and TI. By extension, the functional specialization of dorsal (where) and ventral (what) streams may help us to understand some of the other characteristic features of AVH and TI described in Section 1.

### Abnormal Activation of the Ventral Processing Stream: Perceiving Speaker Identity

One of the most challenging features to explain about AVH or TI is why inserted thoughts and *voices* are often attributed to somebody else (an identity other than the self; see Table 3 –columns). Indeed, this aspect of the phenomenology is particularly difficult to reconcile with the dominant comparator account of either experience, which assumes that AVH or TI are misattributed inner speech: since if this model was correct the identity of the inserted thought or *voice* might be expected to be similar to the self in some fundamental ways. However, this expectation is mostly unmet. In fact, inserted thoughts and *voices* are often perceived or attributed to a different age/gender, individual (known or unknown), or entity with malevolent or benevolent intent (Spirits, the Devil, the government; for a review, see Badcock and Chhabra 2013). For example, “..out of nowhere I heard this woman’s voice in my head whisper to me. She was talking really, really fast, then it was more women talking. I looked everywhere thinking it was maybe the radio? But no, it followed me to the train.” (Angie 2013), or “Thoughts are put into my mind like ‘kill God’. It’s just like my mind working, but it isn’t. They come from this chap, Chris. They’re his thoughts.” (Frith 1992, p 66). Indeed, a recent data synthesis of 100 clinical voice-hearers reported that representations of voice identity (e.g., a family member) could be formulated in 78 % of cases (Corstens and Longden 2013). Furthermore, previous evidence suggests that a recognizably non-self speaking voice is a key feature used to differentiate *voices* from one’s own thoughts, more so than other auditory qualities such as loudness or clarity (Hoffman et al. 2008). Interestingly, however, despite the increased risk of TI in people with AVH, there seem to be no previous studies that have provided a detailed phenomenological comparison of attributed identity in AVH and TI. For example, patients with schizophrenia hear *voices* of both genders but there is a preponderance of adult male *voices* (Corstens and Longden 2013; McCarthy-Jones et al. 2014). Future studies of TI should therefore investigate whether the identity of inserted thoughts is also attributed more often to males than females.

Perhaps perceptions and beliefs about the identity of inserted thoughts or *voices* are meaningless, reflecting idiosyncratic language use related to thought disorder. However, this explanation seems unlikely since these experiences are often reported even when disorganized thinking is not present. Alternatively, assigning names to AVH may reflect a secondary cognitive strategy, rather than an integral part of voice-hearing, simply to keep track of multiple *voices*. However, the ability to assign a separate name seems to rest on the lived experience that the identity of hallucinated *voices* can, in some way, be distinguished (e.g., the older, dominant male *voice* is called ‘the Judge’) as particular, significant agents.^5^ Furthermore, judgments about the physical and social identity (age/gender, dominance/trustworthiness) of *voices* are directly related to the relationship and amount of distress that occurs with AVH (Beavan 2011). The link between identity and distress does not seem to have been examined in relation to TI, but the association in AVH suggests that this aspect of the experience cannot simply be dismissed as irrelevant. Rather, assignment of a name/identity in AVH and TI is likely to be psychologically meaningful, tied to each individuals life’s history and manner of relating to others (Corstens and Longden 2013; Paulik 2012),^6^ and grounded in the basic neural resources involved in the perception and recognition of speaker identity (Badcock and Chhabra 2013).

Our understanding of how the brain recognizes ‘who’ is speaking has improved greatly in recent years (reviewed in: Mathias and von Kriegstein 2014; Schweinberger et al. 2014). For example, in the model proposed by Belin, the analysis of vocal identity occurs in a series of hierarchical stages beginning with the acoustical processing of voices, regardless of familiarity, in the temporal voice areas of the ventral auditory stream. This is followed by processing in modality-dependent voice recognition units, in anterior regions of superior temporal sulcus (STS), which feeds into supramodal ‘person identity nodes’ whereby access to biographical information (such as names) may be gained (Belin et al. 2011; Bethmann and Brechmann 2014). Thus normal processing of voices involves increasingly more abstract representations of speaker identity, independent of other (e.g., acoustic) features (Warren et al. 2006). Current evidence also suggests that individual voices are coded relative to how different they are from a prototypical, or average, voice (i.e., a ‘prior’; Andics et al. 2013; Andics et al. 2010; Latinus et al. 2013), which indicates that both prediction and prediction error signals are routinely implemented in the perception of speaker identity.

Given the salient attributions of identity in many AVH and TI, it seems likely that *both* these symptoms would engage the ventral auditory pathway (for evidence on AVH see: Allen et al. 2012; Badcock 2010; Diederen et al. 2012). Specifically, it might be expected that abnormal activation at lower levels in the hierarchy would be associated with the intrusion of an unfamiliar *voice/*external identity, whilst the intrusion of a familiar *voice* (e.g., the voice of past abuser) or personified thought (e.g., from a man called ‘Chris’) may be associated with dysfunctional activation at increasingly higher levels of the ventral hierarchy. To date, there is a dearth of empirical evidence on voice perception mechanisms in TI. However, there are a growing number of behavioural and neurobiological studies showing impairments in the perception and recognition of voices in clinical and non-clinical *voice* hearers, which suggests that the underlying pathway is dysfunctional (Alba-Ferrara et al. 2012; Badcock and Chhabra 2013). Mou et al. (2013), for instance, used a voice recognition task in a functional imaging paradigm and showed that patients hearing *voices* (compared to those who do not) had reduced connectivity in frontotemporal networks involved in voice identification. However, since their patients were matched on the presence of delusions, the authors also concluded that faulty appraisal of voice identity was specific to AVH (Mou et al. 2013). But the small sample sizes in this study (*N* = 13 per group) tempers these conclusions, and leaves open the possibility that alterations in functional connectivity of the auditory ventral pathway occur in TI as well as AVH (Alba-Ferrara et al. 2012; Chhabra et al. 2012a). Another possibility is that impaired precision of encoding of basic acoustic cues (Chhabra et al. 2012b; 2014; see also: Javitt 2009) generates prediction error signals that propagate to increasingly higher levels of the ventral processing stream.

In short, the APS framework offers a new way of thinking about the sense of external identity in AVH and TI, based on well-established mechanisms for human voice perception. The remainder of this section will consider whether the different spatial attributes of these symptoms can be accommodated in this framework as well (see Table 3 – rows).

### Abnormal Activation of the Dorsal Processing Stream: Perceiving Spatial Location

First person accounts of TI describe experiences that are located *inside* the head (i.e., in personal/internal space) or have been *put into* the head (implying a trajectory in space) from elsewhere.^7^ In contrast, hallucinated *voices* are typically reported as arising from a fixed location in internal or external (extracorporeal) space. Indeed, recent surveys suggest that the location of AVH is about equally likely to be internal or external in clinical and non-clinical voice-hearers (Daalman et al. 2011; McCarthy-Jones et al. 2014), consistent with the view that the perceived location of *voices* may have limited diagnostic utility in patients with psychosis (Longden et al. 2012). Of course, it’s possible that the self-reported localization of these symptoms is simply unreliable. For example, there are conflicting reports of a tendency towards externalization, internalization, or stability of *voice* location over time (McCarthy-Jones et al. 2014; Nayani and David 1996; Plaze et al. 2011). However, if this explanation is correct, it’s far from clear why the subjective location of inserted thoughts is not equally inconstant. Alternatively, the lived experience of AVH and TI in internal or external space might be an accurate representation of the perceptual experience, grounded in atypical activation of mechanisms for spatial localization (Fisher et al. 2012). Moreover, if the combined occurrence of AVH and TI marks a critical tipping point in the transition to psychosis (as noted in section 1.1 above) then it might be important to know much more about developmental changes in auditory spatial processing in healthy young adults who are, or are not, at risk for psychosis (Kuhnle et al. 2013). For instance, McKague and colleagues (McKague et al. 2012) have already shown that healthy young adults predisposed to hallucinations have no difficulty discriminating between internally and externally perceived sounds (see also Badcock et al. 2008). A particular strength of this study was its capacity to examine spatial location independently of speaker identity, making it a potentially valuable task for future studies of at-risk populations experiencing AVH and TI together.

Models of human auditory functioning have identified a dorsal auditory pathway which is primarily involved with processing the spatial features of sounds. For example, functional neuroimaging provides support for a posterior auditory stream which encompasses planum temporale (PT), inferior parietal lobe (IPL) and middle frontal gyrus, which has traditionally been viewed as processing the ‘where’ features of sound (i.e., spatial localization; Ahveninen et al. 2014; Arnott and Alain 2011; Deouell et al. 2007) – though more recent interpretations also suggest this pathway plays a crucial role in linking sounds to actions (and the intentions) that generated them (Arnott and Alain 2011). This latter emphasis provides an obvious point of connection with previous literature linking first rank symptoms to defective self-monitoring of actions and intentions in the IPL and related neural circuits (Frith 2012; Frith et al. 2000; Venkatasubramanian et al. 2011). However, of particular interest here, two recent studies have provided evidence of structural and functional changes in the dorsal auditory pathway associated with the experience of AVH in external space (Looijestijn et al. 2013; Plaze et al. 2011). Looijestijn et al. (2013) found that externally located *voices* were associated with an increased neural response in PT and middle frontal gyrus (though not the IPL) and suggested that internally located AVH may be distinguished from those experienced in external space by their lack of activation in the ‘where’ pathway. If this interpretation is correct, then a lack of activation in dorsal auditory pathway might be predicted for TI as well, given their prominent internal location. However, structural alterations in the temporoparietal junction (TPJ) have also been reported in schizophrenia patients with AVH, leading Plaze and colleagues to conclude that the spatial location of AVH is associated with right TPJ anatomy – “a key region of the ‘where’ auditory pathway” (Plaze et al. 2011, p. 212). Indeed, it’s been proposed that “over-activation of functional modules in the TPJ that subserve social communication produces the syndrome of schizophrenia” (Wible 2012, p. 3). Equivalent studies of TI are rare, but a single case study of a schizophrenia patient capable of reporting the exact onset of inserted thoughts showed that the moment of intrusion was associated with activation in the left supramarginal gyrus (in the dorsal pathway), whilst persistence of TI was associated with abnormal activation in the angular gyrus (Kuhn et al. 2010). Consequently, when considering the data together, an alternative proposal emerges, namely, that the different spatial qualities of AVH and TI may arise from distinct, but overlapping dysfunctions in the dorsal auditory stream (see Table 3).

## Summary and Implications

The evidence presented above suggests that the phenomenological features of both TI and AVH can be connected to dysfunctional activation of auditory processing streams. Importantly, within this framework, neither pathway alone is sufficient to understand the quality of these experiences. Rather, the phenomenological complexity of these symptoms is related to the combination of activity, at different hierarchical stages, within each separable auditory stream. Combinatorial accounts of hallucinated *voices* have become more common in recent years as a result of the increasing recognition that single (cognitive or neural) mechanisms alone fail to explain the heterogeneous nature of the experience (Stephane 2013; Waters et al. 2006). A similar line of thinking has recently been applied to distinguish inserted thoughts from own thoughts, intrusive thoughts or communicated thoughts, in terms of a combination of disturbance in control and identity (referred to as authorship) (Vosgerau and Voss 2014), which resonates with the neuropsychological approach presented here. In order to test these ideas experimentally, it will be important for future research to directly compare the cognitive and neural basis of inserted/intrusive thoughts and AVH in patients (e.g., with psychosis or obsessive compulsive disorder) and non-patient groups. Importantly, if these ideas are correct they may rule out accounts of AVH and TI as ordinary intrusive thoughts that have somehow been misattributed (see also Vicente 2014). Furthermore, as Table 3 shows, when viewed within the APS framework, hallucinatory *voices* can be seen to capture a broader range of features – and corresponding mechanisms – than inserted thoughts. Interestingly, this parallels recent conclusions from factor analytic research (Peralta et al. 2013), and points to a mechanistic basis for the marked difference in prevalence between these symptoms found in epidemiological surveys. Conversely, the presence of at least some degree of overlap in dorsal and ventral dysfunction may partly account for the tendency for AVH and TI to co-occur (Nayani and David 1996; Smeets et al. 2013).

The APS framework bridges the biology and phenomenology of AVH and TI by taking advantage of the best available evidence on normal voice perception. It also complements and extends existing hierarchical predictive coding accounts of psychotic symptoms (Adams et al. 2013; Fletcher and Frith 2009), by identifying potential stages/sites of prediction error signals in dorsal and ventral auditory streams. Undoubtedly, the framework sketched out here is just the beginning. Most notably, the role of identity and location in AVH and TI was highlighted, but the content (words/thoughts) and emotions conveyed in these experiences were not addressed. However, the dorsal-ventral architecture of human perception may offer a more general template for understanding hallucinations in other modalities as well as a range of other anomalous experiences (see Tables 4 and 5).

**Table 4:** A multi-dimensional approach to self-disturbance based on body perception

Spatial Location (Dorsal)	Identity (Ventral)	
	Self	Other
Internal location	Perception of body parts	Somatic/tactile hallucinations
External location	Out-of-body experiences	Sensed presence

**Table 5:** A multi-dimensional approach to self-disturbance based on action perception

Spatial Location (Dorsal)	Identity (Ventral)	
	Self	Other
Internal location	Perception of biological motion/action	Delusions of control [by unseen/proximal forces] Replaced will
External location	Delusional influence	Delusional sense of being manipulated [by unseen/ distant forces].

A number of significant gaps in the literature are worth mentioning, not least of which is the dearth of empirical studies specifically focused on the nature and experience of inserted thoughts. In addition, although the phenomenological assessment of AVH is well-served with a range of detailed assessment tools (Ratcliff et al. 2011) such precision is missing in the assessment of TI. A major corollary of the ideas presented in this paper, is that a much more careful approach to recruitment will be required in future research, in order to match participants on specific phenomenological characteristics (e.g., to examine whether inserted and hallucinated *voices* have the same functional basis). Consequently, the development of sensitive and standardized measures for the assessment of TI will be central to this endeavor. Such tools will also be vital to comparing the experience of TI in clinical and non-clinical populations. If the combined presence of AVH and TI is critical in the emergence of psychosis (Smeets et al. 2013) then a challenge for the future will be to understand the developmental trajectory of ventral and auditory pathways in healthy individuals with TI and AVH who do, and do not, go on to develop psychosis.

There are also a number of limitations in this paper. For example, the focus on dysfunctional activation *within* each auditory pathway is informative but fails to consider the dynamic interactions *between* dorsal and ventral streams. Importantly, there is increasing evidence that different kinds of social and environmental adversity may be associated with different kinds of symptoms (Bentall et al. 2014; Wickham et al. 2014) but the influence of these factors in the APS framework was not addressed. Similarly the influence of genetic and molecular mechanisms on symptom development and auditory function was not explored (Hugdahl et al. 2015). On the other hand, situating AVH and TI within a common framework may have significant clinical implications for treatment of these symptoms. For instance, exploring the different subjective characteristics of each experience and drawing connections with normal perceptual processes may provide new insights for people having these experiences and improve the therapeutic alliance between patient and clinician (Laroi 2006). Furthermore, a better understanding of the cognitive and neural basis of AVH and TI may help in the development of common and distinctive treatment strategies - in keeping with the move towards “precision medicine” in mental health (Insel 2011). For example, if hallucinated *voices* and inserted thoughts share a similar disturbance in executive control then both may benefit from specific treatments targeting this process. Recent evidence suggests, for instance, that transcranial direct current stimulation could be useful for modulating the process of reality filtering (Manuel et al. 2014), offering a potentially new approach to the treatment of both AVH and TI.

## Electronic supplementary material

Below is the link to the electronic supplementary material.

supplementary Table 1 (DOCX 63 kb)

## Footnotes

1 For brevity, I use *voices* (in italics) to refer the perception of voices in the absence of external stimulation and AVH to refer to all forms of auditory verbal hallucinations.

2 From this perspective, the inner speech/self-monitoring account of AVH represents a special case of predictive coding which allows us to predict the sensory consequences of our own actions.

3 Indeed, recent electrophysiological evidence suggests this capacity can dissociated from the ability to know which episode in the past a memory refers to (Liverani et al. 2015).

4 Note – this may equate to saying that AVH and TI do not “feel” like memories.

5 For a more detailed discussion of the function of proper names, see Jeshion (2009).

6 Note, this means that the names/identities reported in AVH and TI need not be limited to individuals who have been directly perceived or interacted with in the past.

7 Humpston and Broome (2015) describe TI as: “an experience of someone or something else’s thoughts *penetrating into* one’s head from the external” (p.7; emphasis added).
