# Supplementary material for: A Neuropsychological Approach to Auditory Verbal Hallucinations and Thought Insertion - Grounded in Normal Voice Perception
Source: Rev Philos Psychol. 2015 Jun 4;7(3):631–52. doi: 10.1007/s13164-015-0270-3 (PMC4995233; doi:10.1007/s13164-015-0270-3)
Supplement: Supplementary file 1 — (DOCX 63 kb) [file 13164_2015_270_MOESM1_ESM.docx]

| **Supplementary Tables**  **Table 1a: Thought insertion and auditory verbal hallucinations (present state)** | | | | |
| --- | --- | --- | --- | --- |
|  | | | | |
|  | | **Auditory verbal hallucinations** | | Total |
|  |  | Present state: no | Present state: yes |  |
| **Thought insertion** | Present state: no | 1236 | 362 | 1598 |
|  | Present state: yes | 77 | 150 | 227 |
| Total | | 1313 | 512 | 1825 |

| **Table 1b: Thought insertion and auditory verbal hallucinations (lifetime)** | | | | |
| --- | --- | --- | --- | --- |
|  | | | | |
|  | | **Auditory verbal hallucinations** | | Total |
|  |  | Lifetime: no | Lifetime: yes |  |
| **Thought insertion** | Lifetime: no | 606 | 698 | 1304 |
|  | Lifetime: yes | 112 | 409 | 521 |
| Total | | 718 | 1107 | 1825 |
